# Supplementary material for: Metal–Site Dispersed Zinc–Chromium Oxide Derived from Chromate–Intercalated Layered Hydroxide for Highly Selective Propane Dehydrogenation
Source: Molecules. 2024 Jun 27;29(13):3063. doi: 10.3390/molecules29133063 (PMC11243157; doi:10.3390/molecules29133063)
Supplement: Supplementary file 1 [file molecules-29-03063-s001.zip › molecules-3031327-supplementary.pdf]

Supplementary Materials

Metal-Site Dispersed Zinc-Chromium Oxide Derived from Chromate-Intercalated Layered Hydroxide for Highly Selective Propane Dehydrogenation

Lu Xue [1], Maoqi Pang [1], Zijian Yuan [1] and Daojin Zhou [1,*]

1 State Key Laboratory of Chemical Resource Engineering, Beijing University of Chemical Technology, Bei-jing, China

* Correspondence: zhoudj@mail.buct.edu.cn

CONTENTS

Supplementary Materials 1

CONTENTS 2

Figure S1. TEM of (a) Zn_1Cr_2-CO_3^{2-} and (b) Zn_1Cr_2-CrO_4^{2-} precursors. 3

Figure S2. Raman spectrum of the Zn_1Cr_2-$CO_3^{2-}$and Zn_1Cr_2-CrO_4^{2-} precursors. 3

Figure S3. XPS spectra of Cr 2p over the Zn_1Cr_2-CrO_4^{2-} precursor. 3

Figure S4. XRD patterns of (a) different compositions of ZnCr-CrO_4^{2-}—MMO and (b) Zn_1Cr_2-CrO_4^{2-} calcined at different temperatures. 4

Figure S5. TEM of (a) Zn_1Cr_2-CO_3^{2-}-MMO and (b) Zn_1Cr_2-CrO_4^{2-}-MMO. 4

Figure S6 (a) Initial propane conversion and (b) propylene selectivity of Mg_2Cr_1-CrO_4^{2-}-MMO, Zn_1C_{r2}-CO_3^{2-}-MMO and Zn_1Cr_2-CrO_4^{2-}-MMO (Reaction conditions: 550 °C, WHSV = 22.3 h^{-1}, C_3H_8:N_2 = 1:4.32). 5

Figure S7. (a) Propane conversion and (b) propylene selectivity of Zn_1Cr_2-CO_3^{2-}-MMO and Zn_1Cr_2-CrO_4^{2-}-MMO (Reaction conditions: 550 °C, WHSV = 22.3 h^{-1}, C_3H_8:N_2 = 1:4.32). (c) Propane conversion and (d) propylene selectivity of Zn_1Cr_2-CrO_4^{2-}-MMO with different WHSV. 5

Figure S8. (a) NH_3-TPD and (b) Py-IR of different proposition ZnCr-CrO_4^{2-}-MMO. 6

Figure S9. XPS spectra of Cr 2p over Zn_1Cr_2-CO_3^{2-}-MMO after reaction. 6

Figure S10. XRD patterns of Zn_1Cr_2-CO_3^{2-}-MMO (a) and Zn_1Cr_2-CrO_4^{2-}-MMO (b) after reaction. 6

Figure S11. TEM of Zn_1Cr_2-CO_3^{2-}-MMO (a) and Zn_1Cr_2-CrO_4^{2-}-MMO (b) after reaction. 7

Figure S12. TGA of Zn_1Cr_2-CO_3^{2-}-MMO and Zn_1Cr_2-CrO_4^{2-}-MMO after reaction. 7

Table S1. Comparison of catalytic performance in this work with previous works. 8

Table S2. NH_3 desorption property of the catalysts. 9

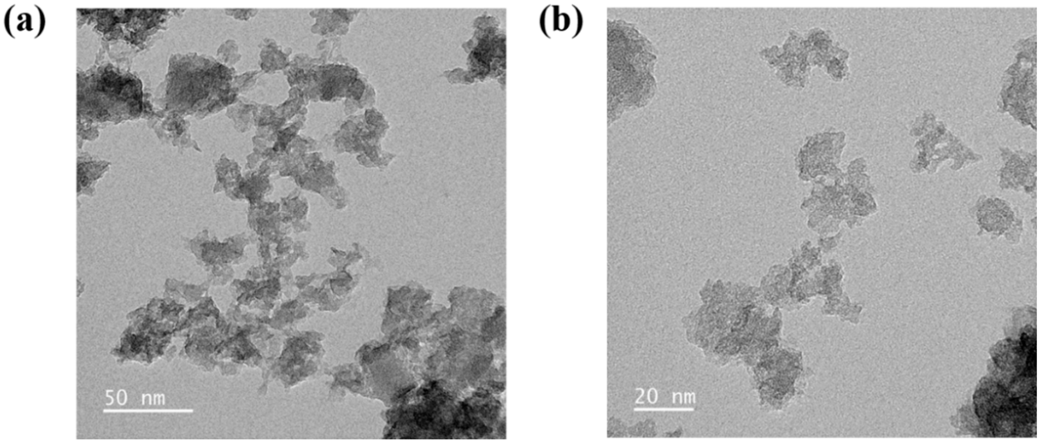

Figure S1. TEM of (a) Zn_1Cr_2-CO_3^{2-} and (b) Zn_1Cr_2-CrO_4^{2-} precursors.

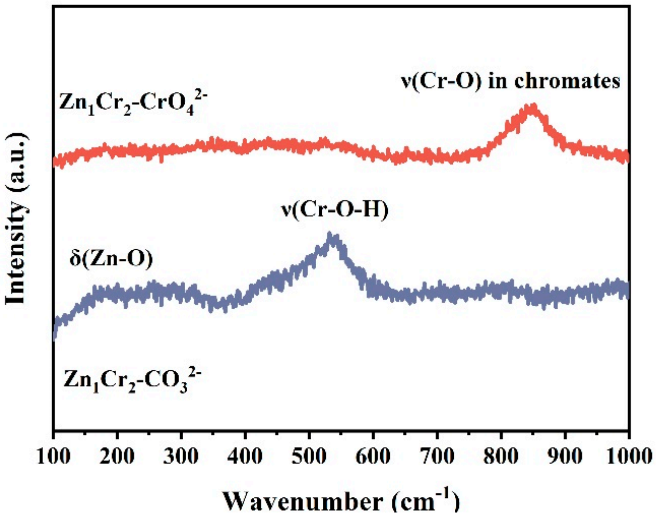

Figure S2. Raman spectrum of the Zn_1Cr_2-$CO_3^{2-}$and Zn_1Cr_2-CrO_4^{2-} precursors.

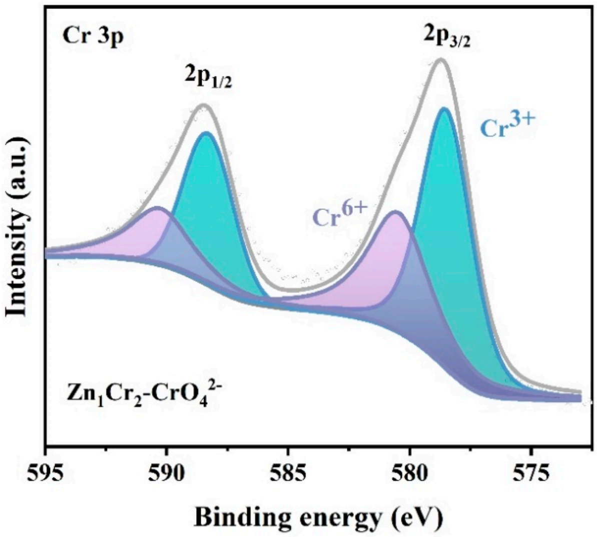

Figure S3. XPS spectra of Cr 2p over the Zn_1Cr_2-CrO_4^{2-} precursor.

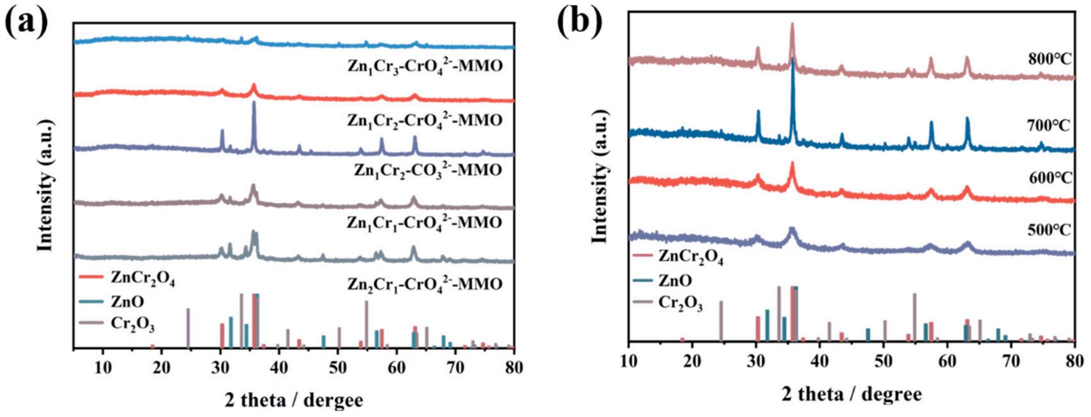

Figure S4. XRD patterns of (a) different compositions of ZnCr-CrO_4^{2-}—MMO and (b) Zn_1Cr_2-CrO_4^{2-} calcined at different temperatures.

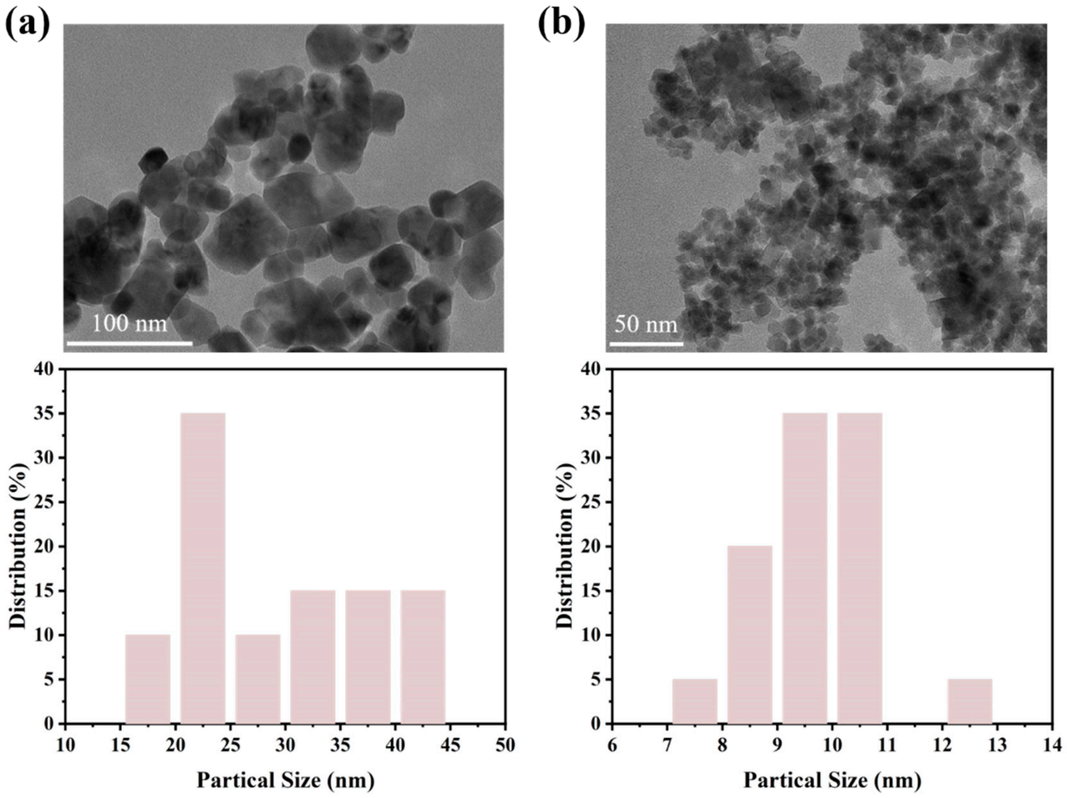

Figure S5. TEM of (a) Zn_1Cr_2-CO_3^{2-}-MMO and (b) Zn_1Cr_2-CrO_4^{2-}-MMO.

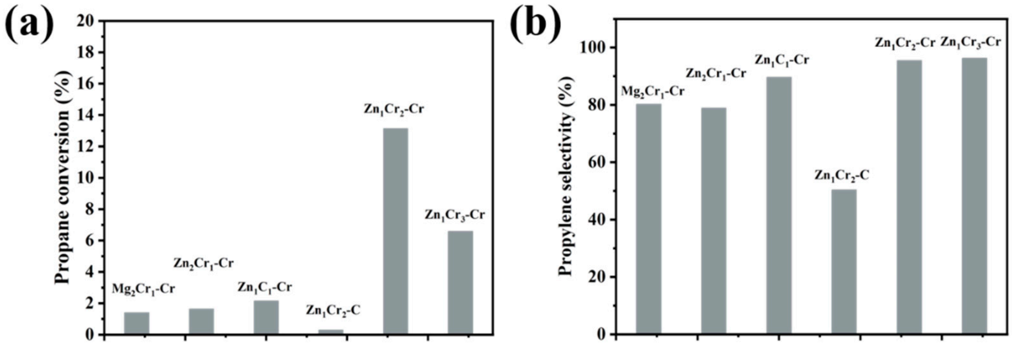

Figure S6 (a) Initial propane conversion and (b) propylene selectivity of Mg_2Cr_1-CrO_4^{2-}-MMO, Zn_1C_{r2}-CO_3^{2-}-MMO and Zn_1Cr_2-CrO_4^{2-}-MMO (Reaction conditions: 550 °C, WHSV = 22.3 h^{-1}, C_3H_8:N_2 = 1:4.32).

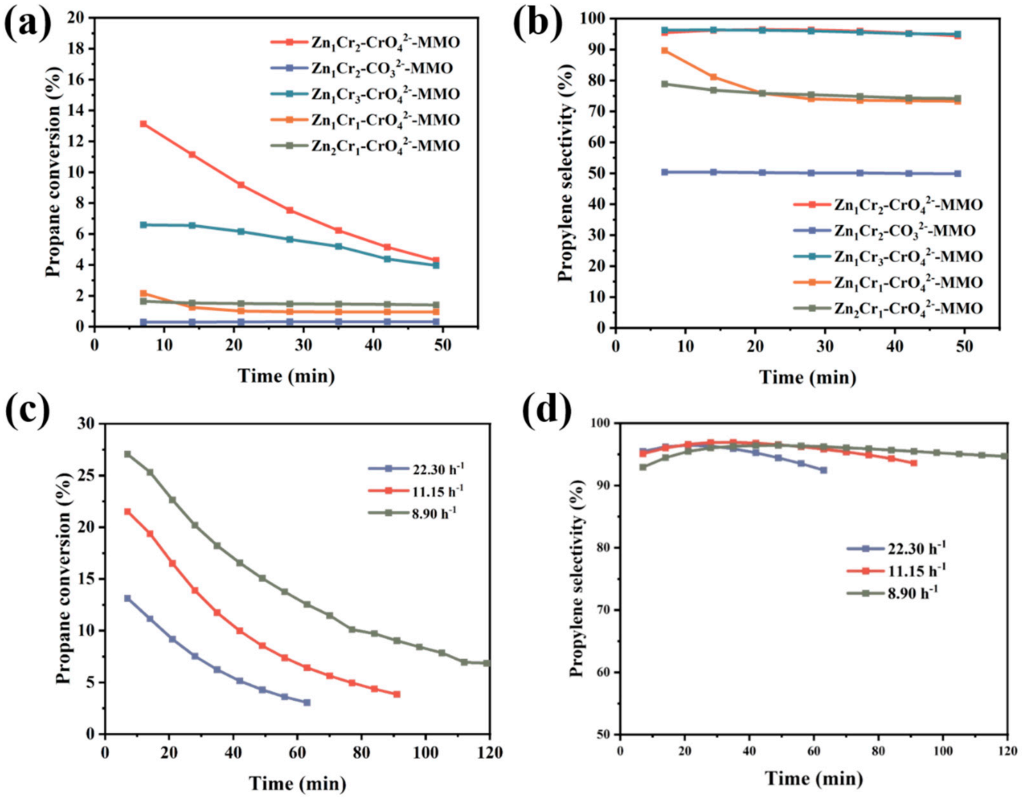

Figure S7. (a) Propane conversion and (b) propylene selectivity of Zn_1Cr_2-CO_3^{2-}-MMO and Zn_1Cr_2-CrO_4^{2-}-MMO (Reaction conditions: 550 °C, WHSV = 22.3 h^{-1}, C_3H_8:N_2 = 1:4.32). (c) Propane conversion and (d) propylene selectivity of Zn_1Cr_2-CrO_4^{2-}-MMO with different WHSV.

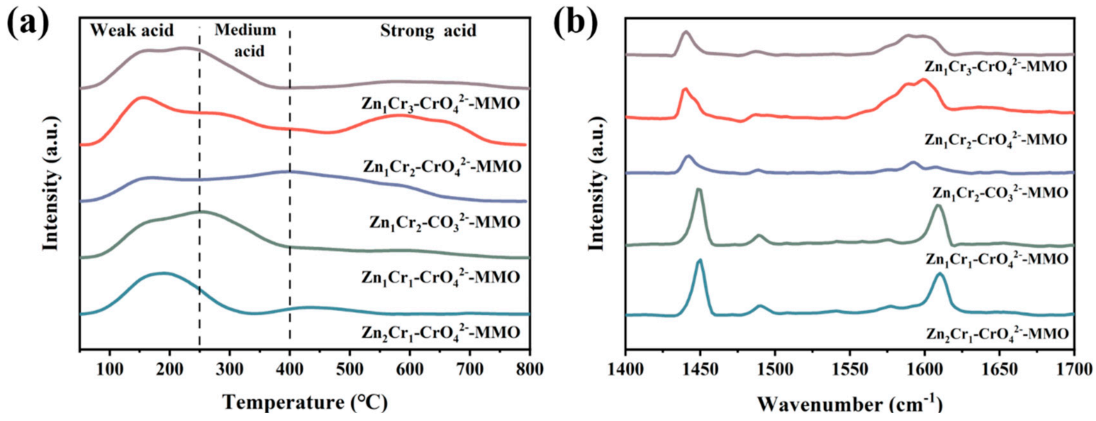

Figure S8. (a) NH_3-TPD and (b) Py-IR of different proposition ZnCr-CrO_4^{2-}-MMO.

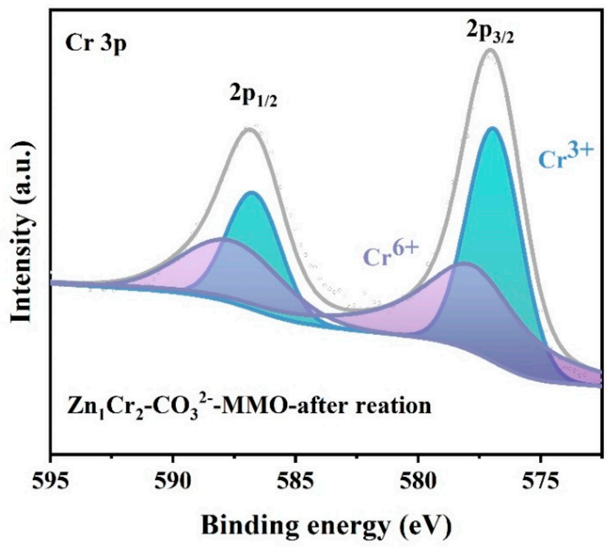

Figure S9. XPS spectra of Cr 2p over Zn_1Cr_2-CO_3^{2-}-MMO after reaction.

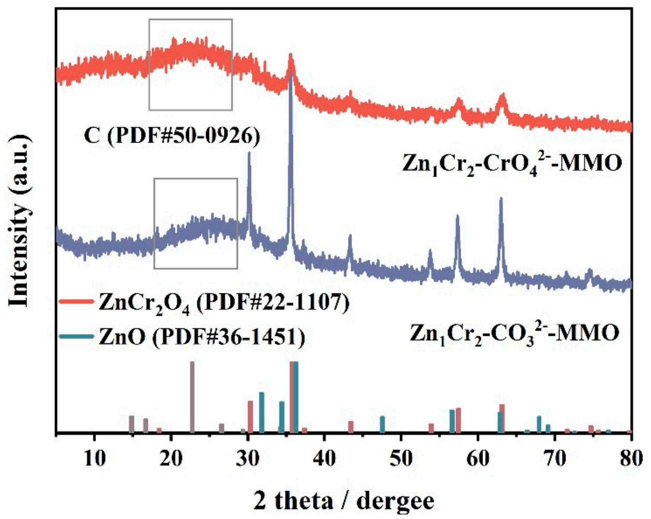

Figure S10. XRD patterns of Zn_1Cr_2-CO_3^{2-}-MMO (a) and Zn_1Cr_2-CrO_4^{2-}-MMO (b) after reaction.

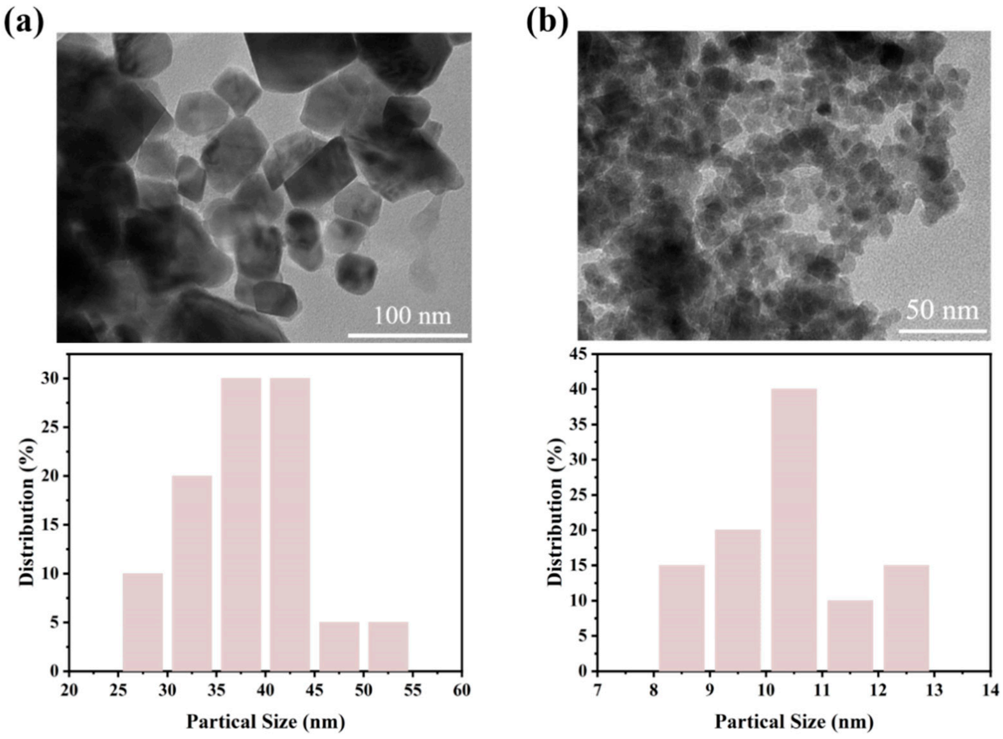

Figure S11. TEM of Zn_1Cr_2-CO_3^{2-}-MMO (a) and Zn_1Cr_2-CrO_4^{2-}-MMO (b) after reaction.

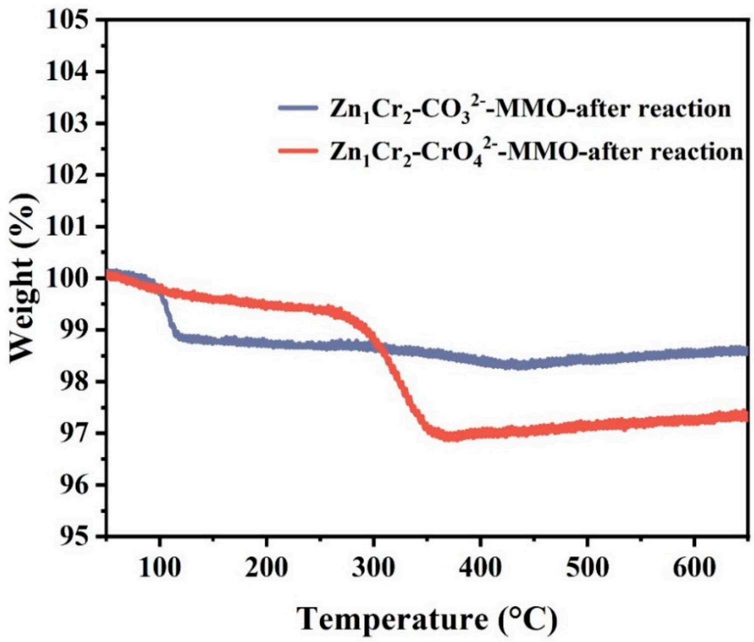

Figure S12. TGA of Zn_1Cr_2-CO_3^{2-}-MMO and Zn_1Cr_2-CrO_4^{2-}-MMO after reaction.

Table S1. Comparison of catalytic performance in this work with previous works.

Catalysis	Temperature(°C)	WHSV	Feed composition	Conversion (%)	Selectivity (%)	Ref
Co-Al_2O_3	590	$2.9h^{-1}$	C_3H_8:H_2:N_2 =1:0.8:3.2	24.8	＞97.1	[40]
0.5%Co@Si-BEA	590	3.66 h^{-1}	C_3H_8:H_2:N_2 =5:4:5	25	＞93	[41]
Mo_2N	500	/	10 vol% C_3H_8in Ar	~11	95	[37]
Co SAs/SiO_2 NMs	550	2.9 h^{-1}	C_3H_8: N_2 = 2:6.4	25	95	[42]
Na-Sn-Beta-30	630	/	20 mol % C_3H_8/80 mol % N_2	40	92	[38]
VO_x/Al_2O_3	600	3 h^{-1}	28 vol% C_3H_8, 28% H_2 in N_2	32	94	[43]
Cr-Al-800	600	/	/	33.2	90.4	[31]
Zn-4@S-1	600	2.4 h^{-1}	10% C_3H_8 balanced by He	41	97	[44]
$Zn_{0.3}$Cr	480	5000 ml $h^{-1}$$gcat^{-1}$	C_3H_8:Ar = 5:95.	31.3	94	[45]
$CrZrO_x$/SiO_2	550	34.5 h^{-1}	40vol% C_3H_8 in N_2	30	85(C=30)	[46]
Rh0.5Sn3.0	600	$10.8h^{-1}$	H_2:N_2:C_3H_8 = 1:1:1	30.8	96.3	[47]
VO_x/ZrO_2	550	$2.07h^{-1}$	C_3H_8:N_2:H_2 =7:36:7	~25	>80	[48]
CoO_x@NC/S-1	600	$1.5h^{-1}$	C_3H_8:N_2 = 5:19	40	>97	[49]
CrO_x/HZSM-5	/	$0.59h^{-1}$	C_3H_8:N_2 = 5:95	~32.6	~94.2	[32]
Zn_1Cr_2-Cr-MMO	550	$8.9h^{-1}$	C_3H_8:N_2=1:4.32	27	>90	This work

Table S2. NH_3 desorption property of the catalysts.

Catalyst	Weak acid site (mmol/g)	Medium acid Site (mmol/g)	Strong acid site (mmol/g)	Total acid site (mmol/g)
Zn_2Cr_1-CrO_4^{2-}-MMO	0.1847	0.0354	0.0295	0.2496
Zn_1Cr_1-CrO_4^{2-}-MMO	0.1954	0.1662	0.0824	0.4440
Zn_1Cr_2-CO_3^{2-}-MMO	0.1185	0.1450	0.1978	0.4613
Zn_1Cr_2-CrO_4^{2-}-MMO	0.2157	0.1396	0.2503	0.6056
Zn_1Cr_3-CrO_4^{2-}-MMO	0.2024	0.0880	0.0567	0.3471
